# Removal of Biomass and Nutrients by Weeds and Direct-Seeded Rice under Conservation Agriculture in Light-Textured Soils of North-Western India

**DOI:** 10.3390/plants10112431

**Published:** 2021-11-11

**Authors:** Salwinder Singh Dhaliwal, Sandeep Sharma, Arvind Kumar Shukla, Vivek Sharma, Makhan Singh Bhullar, Tarundeep Kaur Dhaliwal, Mohammed Alorabi, Saqer S. Alotaibi, Ahmed Gaber, Akbar Hossain

**Affiliations:** 1 Department of Soil Science, Punjab Agricultural University, Ludhiana 141027, India; ssdhaliwal@pau.edu (S.S.D.); sandyagro@pau.edu (S.S.); sharmavivek@pau.edu (V.S.); 2 Indian Institute of Soil Science, Bhopal 462038, India; arvindshukla2k3@yahoo.co.in; 3 Department of Agronomy, Punjab Agricultural University, Ludhiana 141027, India; bhullarms@pau.edu (M.S.B.); tarundhaliwal@pau.edu (T.K.D.); 4 Department of Biotechnology, College of Science, Taif University, P.O. Box 11099, Taif 21944, Saudi Arabia; maorabi@tu.edu.sa (M.A.); saqer@tu.edu.sa (S.S.A.); 5 Department of Biology, College of Science, Taif University, P.O. Box 11099, Taif 21944, Saudi Arabia; 6 Department of Agronomy, Bangladesh Wheat and Maize Research Institute, Dinajpur 5200, Bangladesh

**Keywords:** rice establishment methods, grain and straw yield, weed biomass, removal nutrients, rice and weeds

## Abstract

The escalating scarcity of irrigation water, transplantation of rice on light-textured soils and labour cost acted as major drivers for the transition towards direct-seeded rice (DSR) cultivation from the conventionally flooded transplanting system. Despite these advantages, DSR is a challenge in light texture soil due to heavy weed infestation and a slight decline in crop yield. The weeds compete for nutrients and have an adverse effect on the growth and yield of crops. Hence, to assess the removal of macro and micronutrients by weeds and direct-seeded rice, a field experiment was carried out on sandy loam soil for two consecutive Kharif seasons (2018 and 2019). Three treatments from rice, namely: DSR under zero tillage (DSR-ZT), DSR under conventional tillage (DSR-CT) and DSR under reduced tillage (DSR-RT) were taken as main plots with three tillage treatments in wheat, namely: Conventional tillage without rice straw (CTW-R), Zero tillage without rice straw (ZTW-R) and Zero tillage with straw as mulch using Happy Seeder (ZTW+R) as subplots, replicated thrice. Among the rice establishment methods, DSR-RT showed an edge in terms of rice grain and straw yield (6.18 and 8.14 Mg ha^−1^, respectively) as well as macro- and micronutrient uptake by rice. Under management practices, ZTW+R proved as an efficient strategy in terms of yield and nutrient uptake by crops. The contribution of weeds towards biomass production was maximum under the ZTW-R (9.44%) treatment followed by DSR-ZT (7.72%). The nutrient budgeting showed that macro- and micronutrient removal by weeds was minimum under reduced tillage (24.51 and 50.35%, respectively), whereas it was 21.88 and 44.87% when wheat was grown under conventional tillage without rice straw. In overall, the research study concluded that weeds on an average remove 25.65 % macronutrients (N, P, K) and 51.47% of micronutrients (Zn, Cu, Fe and Mn) in DSR under rice-wheat cropping system.

## 1. Introduction

Rice (*Oryza sativa* L.) serves as the most popular staple food for more than half of the global population. The production of rice acts as the mainstay of the agricultural sector as it accounts for a major share in tropical and subtropical countries including India, being the second-largest producer and consumer in the world [1]. With the growing population burden, sufficient production of rice on limited arable land has now become a priority for developing countries. Numerous aspects that facilitate the accomplishment of the task involves crop genetic improvement, management optimization and socioeconomic factors. Water scarcity has become a major concern for conventional rice cultivation due to the high consumption of water during irrigation [2]. Thus, the development of rice cultivation technology to suppress water use and labour demand while maintaining yield potential is the need of the hour [3].

Conventionally, puddled rice transplanting operations have been used for wetland rice cultivation with a huge amount of water inputs. Crop establishment steps include preparation of nursery bed, rice seedling raising and uprooting followed by the transplantation into the main field [4]. Thus, these practices are not economical as they require an intense amount of water and labour. In addition, conventional transplanted rice causes environmental concerns due to higher emissions of greenhouse gas (CH_4_ and N_2_O emissions), leading to global warming and climate change [5]. Thus, alternate rice production strategies should be implemented to mitigate toxic gas emissions from rice. In the past few years, the direct-seeded rice (DSR) establishment method has been widely practiced to deal with water and labour scarcity while maintaining sustainable yield. Thus, the adoption of DSR offers certain advantages, such as reduced consumption of irrigation water and time, less labour, climate change mitigation, higher productivity of succeeding crops, etc. [5]. Thus, DSR has been frequently adopted by rice growers particularly in Punjab as DSR has been grown on 115,000 ha in Punjab [6] due to cost-effectiveness. Under the DSR system, the sowing of rice seeds is done directly in the soil where they are to grow, rather than transplanting seedlings. Thus, DSR avoids transplanting shocks, preventing adverse effects on soil physical properties and plough-pan formation, lower the duration of crop maturity, save water, energy, labour, fuel, reduces the production cost and increases yield. In DSR, the seed is placed at the optimum depth of 2–3 cm protected by soil for proper germination.

Despite these recompenses, there exist certain factors that limit the productivity of DSR. Among the major biotic factors that cause economic losses in DSR practised on light-textured soils, weeds are considered as the most severe biological constraints for higher rice production. As DSR grown on sandy loam soils has a higher nutrient requirement as compared to a transplanted crop due to higher plant density and greater production of biomass in the vegetative phase; thus, the crop tends to develop nutrient deficiency at the reproductive stage of growth and senesce earlier [7,8]. The early emergence of weeds along with crop seedlings due to favourable soil conditions results in severe competition between crops and weeds for nutrients, space, and light [9]. It had been reported that the losses caused by weeds are manifold ranging from 50% to complete failure of crops [10]. Another study reported that the actual economic loss due to weeds in 10 major crops in India is about $11 billion out of which 21.4% loss is due to weeds in DSR [11]. The uptake of nutrients by weeds in the unweeded plot can be up to nine times higher than in the weed control fields. To date, numerous approaches have been employed for the management of weeds in conservation agriculture systems, including preventive measures, cultural practices, use of herbicides and herbicide-tolerant cultivars. The prime aim of sustainable weed management is to create an environment in the field that naturally suppresses weed emergence and thus minimizes crop–weed competition, rather than merely controlling weeds in the field [12]. The optimization of management practices is mandatory to control weeds during rice production. Tillage systems are of crucial importance for sustainable agricultural production and are well-known traditional means for weed management. Today, different tillage practices, such as conventional tillage, zero tillage, reduced tillage, conservation tillage, etc., have a significant effect on the crop productivity due to the emergence, density and distribution of weed seed in the soil. Naz et al. [13] demonstrated that crop establishment methods, viz. puddling, zero tillage and dry seeding significantly affected the productivity and nutrient uptake in direct-seeded rice in the presence of weeds. Apart from tillage practices, retention of crop residue on the soil surface strongly influences the germination and seedling emergence of weeds due to physical resistance and interference with sunlight availability, as well as improving soil and moisture conservation. Previous studies revealed that retention of crop residue positively affected the wheat yield and decreased the weed density to a significant extent [14]. The weed biomass has a higher content of macro- and micronutrients than the crop plants and absorb more nutrients. Among different rice establishment methods, limited information is available about the removal of macro- and micronutrients by weeds and rice crop separately. Thus, the experiment was carried out to compare the rice yield, macro- and micronutrient budgeting under different crop establishment methods and tillage and residue management practices.

## 2. Materials and Methods

### 2.1. Site Specification

The experiment was carried out during the Kharif seasons (June–October) of 2018 and 2019 at the farm research area of Punjab Agricultural University, Ludhiana (Northwestern India) to know the performance of weed and direct-seeded rice during the removal of biomass and nutrients. The experimental site is located at an elevation of 247 m above mean sea level and lies at 30°54′ latitude and 75°40′ longitude, which represents the central agro-climatic zone of Punjab under the Trans-Gangetic agro-climatic zone of India. The surface soil layer (0–15 cm layer) at the start of the experiment was non-saline (0.36 dS m^−1^) and slightly alkaline having a sandy loam texture (Typic Ustochrept). The pH of soil was 7.86 [15] and contained 4.5 g kg^−1^ Walkley-Black carbon [16], 18.3 mg kg^−1^ 0.5 M NaHCO_3_-extractable P [17] and 114.4 mg kg^−1^ 1N NH_4_OAc-extractable K [15]. The DTPA-extractable Zn, Cu, Fe and Mn in the soil were reported as 0.76, 0.68, 5.86 and 4.55 mg kg^−1^, respectively [18].

### 2.2. Experimental Design and Treatments Details

Different treatments were applied in the rice–wheat system, to examine the efficacy of direct-seeded rice (DSR) under different tillage practices, rice establishment methods and crop residue management practices on removal of macro- (N, P, K) and micronutrients (Zn, Cu, Fe, Mn) by rice crop (grain and straw) and different types of weeds. Rice cultivar (variety PR 115) was taken for DST and sown in the first week of May. In the Rabi season (November–April), wheat variety WH 1105 was sown in the second week of November with seed rate of 100 kg ha^−1^ using the Happy Seeder machine. The experiment was carried out at the Research Farm of the Department of Soil Science, PAU, Ludhiana in a split-plot design with three replications having a plot size of 108 m^2^ (5.4 × 20.0 m). The details of different treatment combinations are given in Table 1.

### 2.3. Soil and Crop Management Practices

Under DSR-ZT, rice was sown in a single operation using zero-till-fertilizer cum seed drill at 20-cm row spacing. Similarly, under DSR-CT, plots were prepared by 2 harrowings + 1 cultivator + 1 planking followed by dry seeding of rice. All treatments received a uniform dose of recommended N (150 kg N as urea), P (26 kg P as di-ammonium phosphate and K (25 kg K as muriate of potash). Based on soil test reports, the whole of P and K were applied at rice planting in all the plots. However, the fertilizer N in DSR-RT, DSR-CT and DSR-ZT was applied in three equal splits, i.e., at 3, 5 and 9 weeks after sowing. Rice was harvested manually in the second week of October.

During the Rabi season, the recommended N (120 kg ha^−1^) as urea was applied in three equal splits, i.e., at sowing, 3 and 8 weeks after sowing. A basal dose of P (26 kg P ha^−1^ as single super phosphate) and K (25 kg K ha^−1^ as muriate of potash) were applied on all plots at the time of sowing.

### 2.4. Collection and Processing of Rice Grain and Straw Samples

Rice grain and straw yield data were collected for two years from all the triplicate treatments at crop harvest and calculated as Mg ha^−1^. The grain and straw samples were also collected, oven-dried at 65 ℃ for 48 h to a constant weight, grinded in the laboratory with the grinder Digital Model ED-5 manufactured by Thomas Scientific, Swedesboro, NJ, USA, and processed for further analysis.

### 2.5. Collection and Processing of Weed Samples

Weed samples were collected for two years from all plots under DSR-CT, DSR-ZT and DSR-NT treatments (main plots) and CTW (-R), ZTW (-R) and ZTW (+R) treatments (subplots) from three locations from each plot and calculated as Mg ha^−1^. Weed samples were collected in the first week of October before rice harvesting and subsequently weight (to obtain fresh weight), oven-dried at 65 °C to a constant weight and grinded with the grinder Digital Model ED-5 for further analysis.

### 2.6. Estimation of Macronutrients (N, P and K) and Micronutrients (Zn, Cu, Fe and Mn) in Weed, Rice Grain and Straw Samples

The total N content from the weed, rice grain and straw samples was determined by the micro-Kjeldahl method [19]. The total P and K concentrations were determined in triple-acid (HNO_3_:H_2_SO_4_:HClO_4_; 10:3:1) digests using the ammonium molybdate method for P [20] and flame photometry for K [19]. The determination of Zn, Cu, Fe and Mn from weed, rice grain and straw samples was carried out as a method given by Isaac and Kerber [21]. These grounded weeds, rice grain and straw samples were digested in a diacid mixture of HNO_3_ and HClO_4_ (3:1) for analysis of Zn, Cu, Fe and Mn concentrations by using a Varian SpectrAA 220G Graphite Furnace Atomic Absorption Spectrometer manufactured by LabX, Otawa, ON, Canada.

### 2.7. Statistical Analysis

The recorded data were statistically analyzed by using split block design in the statistical package SAS software for two-way analysis of variance (ANOVA). The treatments were compared using the least significant difference at the 5% level of significance. Duncan’s multiple range test (DMRT) was employed to assess the differences between treatment means.

## 3. Results and Discussion

### 3.1. Effect of Direct-Seeded Rice on Grain and Straw Yield of Rice and Weed Biomass under Different Rice Establishment Methods 

The results of two-year data demonstrated that different rice establishment methods, viz., DSR-ZT, DSR-CT and DSR-RT, and tillage and rice straw (TRS) management practices, viz., CTW-R, ZTW-R and ZTW+R significantly affected rice grain yield, but not straw yield (Table 2). The highest grain yield and straw yield under rice establishment methods were recorded under DSR-RT. The trend might be attributed to the higher microbial activity with retention of soil structure under reduced tillage as compared to conventional tillage [22,23]. The plots under zero tillage recorded the highest weed biomass which might have negatively affected the crop yield and hence showed lower yield as compared to the crop grown under reduced tillage. Under management practices, maximum grain and straw yield was observed under ZTW+R. Thus, retention of crop residue under zero tillage showed a maximum positive effect on yield. These findings could be attributed to the enhanced organic matter and microbial activities with the retention of crop residue [24].

Significant differences were observed in the case of weed biomass and its share towards the total biomass of rice (grain + straw) and weeds. Among different rice establishment methods, maximum weed biomass was observed under DSR-ZT (1.19 Mg ha^−1^) followed by DSR-RT (1.14 Mg ha^−1^) and DSR-CT (0.96 Mg ha^−1^). Under TRS management practices, maximum biomass was observed with ZTW-R followed by ZTW+R and CTW-R. The results illustrated that tillage was an effective measure to reduce the occurrence of weeds. The perturbations in soil due to tillage may bury weed seeds in the deep soil layer and thus suppresses the emergence of seedlings. The retention of crop residue resulted in relatively lower weed biomass than that of removal of crop residue. The retention of crop residues might have altered the physicochemical environment of the seed emergence by blocking light or acting as a barrier for the seed germination of weeds [25]. 

The results of our study reported that weeds contributed significantly towards total biomass production (6.0–9.4%) under direct-seeded rice production (Table 2). As such, weeds contributed towards the maximum per cent of total biomass (7.72) in DSR-ZT treatment under different rice establishment methods and tillage. However, under different rice straw management practices, the maximum weed share towards biomass production is obtained in ZTW-R (9.4%), where wheat was sown with zero tillage followed by removal of rice straw.

### 3.2. Removal of Macronutrients (N, P and K) by Rice Grain and Straw 

The highest nitrogen uptake in grain was found under DSR-RT (38.7 kg ha^−1^) followed by DSR-ZT (37.4 kg ha^−1^) and, then, in DSR-CT (35.4 kg ha^−1^) among the rice establishment methods, irrespective of the TRS management practices. A similar trend was observed for P and K uptake in grain. The maximum values for P and K uptake in rice grain recorded were 8.4 and 34.8 kg ha^−1^, respectively. In straw, the maximum N, P and K uptake values were 33.8, 19.4 and 52.4 kg ha^−1^, respectively, under DSR-RT (Table 3). The results can be explained based on fact that intensive tillage intensifies the SOM decomposition, but, for the decomposition, the microorganisms are responsible; therefore, microbial activity can even increase. This can lead to nutrient release in bioavailable form and their sooner losses and soil depletion. [26]. Thus, the plot under DSR-CT showed the least nutrient uptake. The lower P uptake under DSR-ZT as compared to DSR-RT might be related to higher weed–crop competition due to high weed biomass in DSR-ZT.

Under TRS management practices, the N uptake in rice grain and straw was higher in the case of ZTW+R (40.4 and 37.9 kg ha^−1^) with respect to CTW-R and ZTW-R. A similar trend was observed for P and K uptake. For P, the maximum uptake recorded in grain and straw was 11.6 and 22.2 kg ha^−1^ under ZTW+R. Similarly, the K uptake was found significantly higher under ZTW+R with a maximum value of 38.4 and 56.1 kg ha^−1^ in grain and straw (Table 3). The results suggested that zero tillage with retention of crop residue led to higher nutrient uptake in DSR. The results might be attributed to the decomposition soil organic matter, which in turn raised the concentration of plant-available nutrients and hence increased nutrient uptake [27,28].

### 3.3. Removal of Macronutrients (N, P and K) by Weeds

The highest nitrogen uptake by weeds was found under DSR-ZT (12.7 kg ha^−1^) followed by DSR-RT (11.2 kg ha^−1^) and, then, in DSR-CT (9.6 kg ha^−1^) among the rice establishment methods, irrespective of the TRS management practices. The P uptake followed a similar trend as that of the N uptake, as it was observed that the P uptake was higher under DSR-ZT (10.9 kg ha^−1^) followed by DSR-RT followed by DSR-CT, but no significant differences were observed among the different treatments. Similarly, the K uptake was found significantly higher under DSR-ZT (43.8 kg ha^−1^) as compared to DSR-RT and DSR-CT. Among the TRS management practices, the N uptake was higher in the case of ZTW-R (12.2 kg ha^−1^) with respect to CTW-R (11.2 kg ha^−1^) and ZTW+R (10.1 kg ha^−1^). Similarly, the P uptake was observed significantly higher under ZTW-R (11.0 kg ha^−1^) as compared to ZTW+R (10.2 kg ha^−1^) and CTW-R (8.8 kg ha^−1^) treatments (Table 4).

The interaction among the different treatments also showed significant differences. Similarly, the K uptake was observed significantly higher under ZTW-R (48.4 kg ha^−1^) as compared to the ZTW+R (40.3 kg ha^−1^) and CTW-R (34.9 kg ha^−1^) treatments. The trend of macronutrient uptake showed a strong positive correlation with the weed biomass. Similar results have been reported earlier, where the trend of N uptake by weeds was well corroborated with weed biomass [29]. Higher weed biomass led to more weed–crop competition for nutrients which resulted in higher nutrient uptake by weed under ZTW-R. Thus, DSR-CT and CTW-R among rice establishment methods and management practices respectively resulted in the least loss of nutrients due to weeds.

### 3.4. Removal of Micronutrients (Zn, Cu, Fe and Mn) by Rice Grain and Straw 

The Zn uptake by rice grain among rice establishment methods was found higher under DSR-RT (110.6 g ha^−1^) followed by DSR-ZT (96.6 g ha^−1^) followed by DSR-CT (85.4 g ha^−1^). However, the Cu uptake was observed higher under DSR-ZT (29.9 g ha^−1^) followed by DSR-RT (25.7 g ha^−1^) and, then, in DSR-CT (25.3 g ha^−1^). Moreover, the Fe uptake among the rice establishment methods, irrespective of the TRS management practices, was found significantly higher under DSR-RT (290.9 g ha^−1^) followed by DSR-ZT (265.8 g ha^−1^) and, then, in DSR-CT (227.5 g ha^−1^). Similar to the Fe uptake, the Mn uptake was observed higher under DSR-RT (62.7 g ha^−1^) as compared to DSR-ZT (54.5 g ha^−1^) and DSR-CT (43.2 g ha^−1^). The Zn, Fe and Mn uptake in straw showed the same trend (Table 5). The results can be explained based on the combined effect of weed–crop competition and nutrient availability. Under reduced tillage, nutrient availability is high due to lesser soil disturbances than conventional tillage and weed-crop competition is low as compared to zero tillage. The results of nutrient uptake by straw also showed a strong positive correlation with straw yield.

Amongst the TRS management practices, it was observed that uptake of Zn, Fe and Mn was found higher under ZTW+R followed by CTW-R followed by ZTW-R. The retention of crop residue might have increased the soil organic matter which resulted in increased micronutrients solubility and availability for plant uptake by forming chelates [30]. Thus, incorporation of organic residue along with the retention of soil structure with least disturbances enhanced the micronutrient availability to crop which resulted in higher micronutrient uptake by plants.

### 3.5. Removal of Micronutrients (Zn, Cu, Fe and Mn) by Weeds 

Rice establishment systems, as well as TRS management practices, expressed a significant effect on the Zn uptake by weeds. Zinc uptake fluctuated from 465.6 (DSR-CT) to 528.4 (DSR-ZT) g ha^−1^ and from 402.6 (CTW-R) to 552.5 (ZTW-R) g ha^−1^, respectively, between rice establishment systems as well as TRS management practices. The data pertaining to the effect of TRS management practices as well as rice establishment systems did not significantly affect the Cu uptake by weeds. Copper uptake varied from 72.7 (DSR-CT) to 83.2 (DSR-ZT) g ha^−1^ and from 72.7 (CTW-R) to 81.4 (ZTW-R) g ha^−1^ between rice establishment systems as well as TRS management practices. The data reported in Table 6 show that the Fe uptake in weeds ranged between 2682.6 (DSR-CT) to 3268.6 (DSR-ZT) g ha^−1^ and 2138.4 (CTW-R) to 3379.2 (ZTW-R) g ha^−1^ between rice establishment methods and the TRS management practices.

A glance at the data related to the Mn uptake (Table 6) in weeds showed that the Mn content ranged from 442.8 (DSR-CT) to 556.9 (DSR-ZT) g ha^−1^ and from 403.6 (CTW-R) to 568.7 (ZTW-R) g ha^−1^ between rice establishment methods and TRS management practices, respectively. The trend was correlated with weed biomass. As weed biomass was maximum under zero tillage in addition to higher nutrient availability. Thus, maximum micronutrient uptake was found to be highest in DST-ZT. In addition, the highest uptake under ZTW-R over the ZTW+R was due to the higher weed biomass that led to higher micronutrient uptake.

### 3.6. Nutrients Budgeting for Removal of Macro and Micronutrients by Rice and Weeds under Different Rice Establishment Methods 

The scrutiny of data from Table 7 shows that the total macronutrient removal by rice under rice establishment methods was maximum under DSR-RT (187.5 kg ha^−1^) followed by DSR-ZT (174.3 kg ha^−1^) and DSR-CT (163.8 kg ha^−1^). Weeds also compete for macronutrient uptake and showed significant total macronutrient uptake. Maximum uptake by weeds was recorded under DSR-ZT (67.4 kg ha^−1^) followed by DSR-RT (60.9 kg ha^−1^) and DSR-CT (58.8 kg ha^−1^). Under management practices, maximum total macronutrient uptake was recorded in ZTW+R (206.8 kg ha^−1^) followed by CTW-R (192.2 kg ha^−1^) and ZTW-R (165.4 kg ha^−1^).

**Figure 1:**
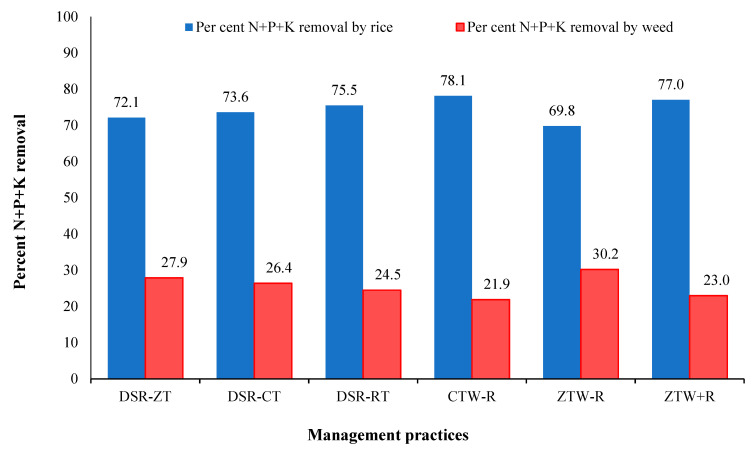
Percentage of macronutrients (N + P + K) removed by rice and weed under different management practices. DSR (Direct-seeded rice), CT (Conventional tillage), RT (Reduced tillage), ZT (Zero tillage), CTW (Conventional till wheat), ZTW (Zero till wheat), R (Rice residue).

Under management practices, the maximum total macronutrient uptake by weeds was recorded in ZTW-R (71.6 kg ha^−1^) followed by ZTW+R (61.7 kg ha^−1^) and CTW-R (53.8 kg ha^−1^). The percent removal of macronutrients by weeds showed that weeds accumulated significantly higher amounts of macronutrients (Figure 1). The maximum contribution of weeds was observed in DSR-ZT (27.9%) followed by DSR-CT (26.4%) and DSR-RT (24.5%). Under management practices, maximum contribution by weeds in macronutrient removal was recorded in ZTW-R (30.2%) followed by ZTW+R (23.0%) and CTW-R (22.9%).

**Figure 2:**
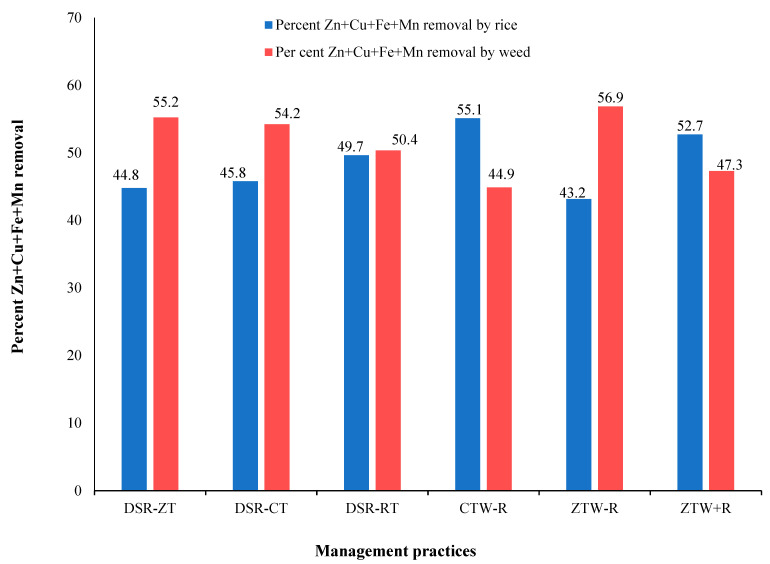
Percentage of micronutrients (Zn + Cu + Fe + Mn) removed by rice and weed under different management practices. DSR (Direct-seeded rice), CT (Conventional tillage), RT (Reduced tillage), ZT (Zero tillage), CTW (Conventional till wheat), ZTW (Zero till wheat), R (Rice residue).

In the case of micronutrients, the total removal by rice under rice establishment methods was maximum under DSR-RT (3796.0 g ha^−1^) followed by DSR-ZT (3597.2 g ha^−1^) and DSR-CT (3092.8 g ha^−1^). Under management practices, maximum total micronutrient uptake was recorded in ZTW+R (3998.1 g ha^−1^) followed by CTW-R (3706.6 g ha^−1^) and ZTW-R (3477.8 g ha^−1^). Weeds also compete for micronutrient uptake as, under rice establishment methods, the maximum uptake was observed under DSR-ZT (4437.1 g ha^−1^) followed by DSR-RT (3849.8 g ha^−1^) and DSR-CT (3663.7 g ha^−1^). Under management practices, maximum total micronutrient uptake was recorded in ZTW-R (4581.8 g ha^−1^) followed by ZTW+R (3586.1 g ha^−1^) and CTW-R (3017.3 g ha^−1^). The percent removal of micronutrients by weeds showed that weeds accumulated significantly higher amounts of micronutrients, even more than rice crops (Figure 2). The maximum contribution of weeds was observed in DSR-ZT (55.2%) followed by DSR-CT (54.2%) and DSR-RT (50.4%). Under management practices, maximum contribution by weeds in macronutrient removal was recorded in ZTW-R (56.9%) followed by ZTW+R (47.3) and CTW-R (44.9%).

## 4. Conclusions

The results of the field study showed that DSR-RT gave the highest grain yield and nutrient removal by grains among rice establishment methods, however, the weed biomass and nutrient removal by weeds were highest in DSR-ZT. Under rice establishment methods, weeds contribute towards 6.0–9.4% of total biomass production. The weeds removed an average of 25.7% and 51.5% of the macro- (N, P, K) and micronutrients (Zn, Cu, Fe, Mn), respectively. Among tillage and straw management, the treatment with zero tillage under removal of crop residue resulted in maximum removal for macro- and micronutrients by weeds as compared to zero tillage with residue and conventional tillage treatments. 

## Figures and Tables

**Table 1:** Description of treatments in the experimental field under rice establishment methods in the rice–wheat cropping system.

Treatments	Methods	Tillage	Wheat Residue	Practice Used during DSR	Spacing (Row to Row)
Rice establishment methods					
DSR-CT	Direct seeded rice	Conventional tillage	Residue of preceding wheat was removed	Tillage for DSR included two passes of disc harrows and two passes of tyne plough followed by planking	20 cm
DSR-ZT	Direct seeded rice	Zero tillage	Residue of preceding wheat was removed	Zero till DSR was sown using inclined plate multi-crop planter	20 cm
DSR-RT	Direct seeded rice	Reduced tillage	Residue of preceding wheat was removed	Zero till unpuddled transplanted rice was mechanically transplanted using self-propelled transplanter in standing water	15–20 cm
Tillage and straw management practices					
CTW (-R)	Two passes of disc and planking	Conventional till wheat	Residue of rice crop was removed	The field was irrigated prior to tyne ploughing. Wheat was sown using the same seed cum fertilizer drill as ZTW-R	20 cm
ZTW (-R)	Direct wheat sowing	Zero till wheat	Residue of rice crop was removed	Wheat was direct-seeded in the no till plots using zero till seed cum fertilizer drill	20 cm
ZTW (+R)	Direct wheat sowing	Zero till wheat	Residue of rice crop was retained	Wheat was direct-seeded into rice residues using a Turbo Happy Seeder v3 model	20 cm

**Table 2:** Yield of the rice grain, straw and weed biomass under different rice establishment methods.

Treatments	Grain Yield of Rice (Mg ha^−1^)	Straw Yield of Rice (Mg ha^−1^)	Weed Biomass (Mg ha^−1^)	Total Biomass (Rice + Weeds) (Mg ha^−1^)	Weeds Share of Total Biomass (%)
Rice establishment methods					
DSR-ZT	6.07 ^ab^	8.06	1.19 ^b^	15.4	7.72
DSR-CT	5.94 ^b^	7.95	0.96 ^c^	14.9	6.46
DSR-RT	6.18 ^a^	8.14	1.14 ^a^	15.5	7.37
LSD (0.05)	0.12	NS	0.04	-	-
TRS management practices					
CTW-R	6.24 ^a^	8.62 ^a^	1.0 ^c^	15.8	6.01
ZTW-R	5.74 ^b^	7.43 ^b^	1.2 ^a^	13.0	9.44
ZTW+R	6.53 ^a^	9.04 ^a^	1.04 ^b^	16.66	6.26
LSD (0.05)	0.37	0.84	0.04	-	-
LSD (0.05) Interactions	NS	NS	0.008	-	-

DSR (Direct-seeded rice), CT (Conventional tillage), RT (Reduced tillage), ZT (Zero tillage), CTW (Conventional till wheat), ZTW (Zero till wheat), R (Rice residue). Averaged values within a column, succeeded by different small letters (a, b, c) differ significantly between different treatments at *p* ≤ 0.05 significance level.

**Table 3:** Removal of macronutrients (kg ha^−1^) by rice grains and straw under different rice establishment methods.

Treatments	Grains			Straw		
	N Uptake	P Uptake	K Uptake	N Uptake	P Uptake	K Uptake
Rice establishment methods						
DSR-ZT	37.4 ^b^	7.8	32.6 ^ab^	30.5 ^b^	17.4 ^b^	48.6 ^b^
DSR-CT	35.4 ^c^	7.2	30.4 ^b^	28.2 ^c^	15.8 ^c^	46.8 ^b^
DSR-RT	38.7 ^a^	8.4	34.8 ^a^	33.8 ^a^	19.4 ^a^	52.4 ^a^
LSD (0.05)	0.6	NS	3.0	2.3	1.6	2.3
TRS management practices						
CTW-R	37.9 ^b^	9.2 ^b^	35.4 ^b^	34.3 ^b^	20.6 ^b^	54.8 ^a^
ZTW-R	36.4 ^b^	7.9 ^b^	30.9 ^c^	28.4 ^c^	16.4 ^c^	45.4 ^b^
ZTW+R	40.4 ^a^	11.6 ^a^	38.4 ^a^	37.9 ^a^	22.4 ^a^	56.1 ^a^
LSD (0.05)	2.5	1.9	2.0	2.6	1.8	2.4
LSD (0.05)Interactions	0.8	1.6	1.7	1.6	1.9	2.1

DSR (Direct-seeded rice), CT (Conventional tillage), RT (Reduced tillage), ZT (Zero tillage), CTW (Conventional till wheat), ZTW (Zero till wheat), R (Rice residue). Averaged values within a column, succeeded by different small letters (a, b, c) differ significantly between different treatments at *p* ≤ 0.05 significance level.

**Table 4:** Removal of macronutrients (kg ha^−1^) by weeds under different rice establishment methods.

Treatments	N Uptake	P Uptake	K Uptake
Rice establishment methods			
DSR-ZT	12.7 ^a^	10.9	43.8 ^a^
DSR-CT	9.6 ^c^	9.5	39.7 ^b^
DSR-RT	11.2 ^b^	9.6	40.1^b^
LSD (0.05)	0.6	NS	3.0
TRS management practices			
CTW-R	10.1 ^c^	8.8 ^b^	34.9 ^c^
ZTW-R	12.2 ^a^	11.0 ^a^	48.4 ^a^
ZTW+R	11.2 ^b^	10.2 ^a^	40.3 ^b^
LSD (0.05)	0.5	0.9	1.0
LSD (0.05) Interactions	0.8	1.6	1.7

DSR (Direct-seeded rice), CT (Conventional tillage), RT (Reduced tillage), ZT (Zero tillage), CTW (Conventional till wheat), ZTW (Zero till wheat), R (Rice residue). Averaged values within a column, succeeded by different small letters (a, b, c) differ significantly between different treatments at *p* ≤ 0.05 significance level.

**Table 5:** Removal of micronutrients (g ha^1^) by rice grain and straw under different rice establishment methods.

Treatments	Rice Grain (g ha^−1^)				Rice Straw (g ha^−1^)			
	Zn	Cu	Fe	Mn	Zn	Cu	Fe	Mn
Rice establishment methods								
DSR-ZT	96.6 ^b^	25.7 ^b^	265.8 ^b^	54.5 ^b^	483.5 ^a^	22.9 ^a^	2432.8 ^a^	215.4 ^b^
DSR-CT	85.4 ^c^	25.3 ^b^	227.5 ^c^	43.2 ^c^	425.4 ^b^	19.4 ^b^	1953.7 ^b^	312.9 ^a^
DSR-RT	110.6 ^a^	29.9 ^a^	290.9 ^a^	62.7 ^a^	487.2 ^a^	24.7 ^a^	2441.9 ^a^	348.1 ^a^
LSD (0.05)	2.23	2.56	13.5	7.2	35.2	2.5	289.1	32.6
TRS management practices								
CTW-R	95.5 ^b^	25.6 ^b^	276.5 ^b^	52.9 ^b^	445.3 ^b^	25.6 ^ab^	2461.7 ^ab^	323.5 ^b^
ZTW-R	90.7 ^b^	26.9 ^a^	258.7 ^c^	51.1 ^b^	425.4 ^c^	26.5 ^a^	2280.8 ^b^	317.7 ^c^
ZTW+R	107.2 ^a^	24.1 ^c^	290.5 ^a^	62.8 ^a^	490.3 ^a^	24.9 ^b^	2663.4 ^a^	334.9 ^a^
LSD (0.05)	6.7	0.9	10.8	4.2	18.3	0.4	301.1	4.4
LSD (0.05) Interactions	NS	NS	NS	NS	NS	NS	NS	NS

DSR (Direct-seeded rice), CT (Conventional tillage), RT (Reduced tillage), ZT (Zero tillage), CTW (Conventional till wheat), ZTW (Zero till wheat), R (Rice residue). Averaged values within a column, succeeded by different small letters (a, b, c) differ significantly between different treatments at *p* ≤ 0.05 significance level.

**Table 6:** Removal of micronutrients (g ha^1^) by weeds under different rice establishment methods.

Treatments	Zn	Cu	Fe	Mn
Rice establishment methods				
DSR-ZT	528.4 ^a^	83.2 ^a^	3268.6 ^a^	556.9 ^a^
DSR-CT	465.6 ^c^	72.7 ^b^	2682.6 ^c^	442.8 ^c^
DSR-RT	497.6 ^b^	80.7 ^ab^	2756.7 ^b^	514.8 ^b^
LSD (0.05)	12.9	9.4	37.7	28.6
TRS management practices				
CTW-R	402.6 ^c^	72.7 ^b^	2138.4 ^c^	403.6 ^c^
ZTW-R	552.5 ^a^	81.4 ^a^	3379.2 ^a^	568.7 ^a^
ZTW+R	452.6 ^b^	76.4 ^ab^	2573.6 ^b^	483.5 ^b^
LSD (0.05)	35.6	6.4	81.3	58.5
LSD (0.05) Interactions	NS	NS	NS	NS

DSR (Direct-seeded rice), CT (Conventional tillage), RT (Reduced tillage), ZT (Zero tillage), CTW (Conventional till wheat), ZTW (Zero till wheat), R (Rice residue). Averaged values within a column, succeeded by different small letters (a, b, c) differ significantly between different treatments at *p* ≤ 0.05 significance level.

**Table 7:** Nutrients removal by rice grain + straw and weed biomass and budgeting under different rice establishment methods.

Treatments	N + P + K Removed by Rice Grain + Straw	N + P + K Removed by Weeds	N + P + K Removed by Weeds	Zn + Cu + Fe + Mn Removed by Rice Grain + Straw	Zn + Cu + Fe + Mn Removed by Weeds	Zn + Cu + Fe + Mn Removed by Weeds
	kg ha^−1^		%	g ha^−1^		%
Rice establishment methods						
DSR-ZT	174.3	67.4	27.9	3597.2	4437.1	55.2
DSR-CT	163.8	58.8	26.4	3092.8	3663.7	54.2
DSR-RT	187.5	60.9	24.5	3796.0	3849.	50.4
Mean	175.2	62.4	26.3	3495.3	3983.5	53.3
TRS management practices						
CTW-R	192.2	53.8	21.9	3706.6	3017.3	44.9
ZTW-R	165.4	71.6	30.2	3477.8	4581.8	56.9
ZTW+R	206.8	61.7	23.0	3998.1	3586.1	47.3
Mean	188.2	62.4	25.0	3727.5	3728.4	49.7
Overall Mean	181.7	62.4	25.7	3611.4	3855.9	51.5

DSR (Direct-seeded rice), CT (Conventional tillage), RT (Reduced tillage), ZT (Zero tillage), CTW (Conventional till wheat), ZTW (Zero till wheat), R (Rice residue).

## Data Availability

All data are available in the manuscripts.
